# Preparation of nanoliposomes containing HER2/neu (P5+435) peptide and evaluation of their immune responses and anti-tumoral effects as a prophylactic vaccine against breast cancer

**DOI:** 10.1371/journal.pone.0243550

**Published:** 2020-12-10

**Authors:** Laleh Naghibi, Mona Yazdani, Amir Abbas Momtazi-Borojeni, Atefeh Razazan, Sheida Shariat, Mercedeh Mansourian, Atefeh Arab, Nastaran Barati, Mahdieh Arabsalmani, Azam Abbasi, Zahra Saberi, Ali Badiee, Seyed Amir Jalali, Mahmoud Reza Jaafari

**Affiliations:** 1 Nanotechnology Research Center, Pharmaceutical Technology Institute, Mashhad University of Medical Sciences, Mashhad, Iran; 2 Department of Pharmaceutical Nanotechnology, School of Pharmacy, Mashhad University of Medical Science, Mashhad, Iran; 3 Department of Medical Biotechnology, Faculty of Medicine, Mashhad University of Medical Sciences, Mashhad, Iran; 4 Department of Molecular Medicine, School of Advanced Technologies in Medicine, Tehran University of Medical Sciences, Tehran, Iran; 5 Biotechnology Research Center, Pharmaceutical Technology Institute, Mashhad University of Medical Sciences, Mashhad, Iran; 6 Vice Chancellor for Research and Technology, Hamadan University of Medical Science, Hamadan, Iran; 7 Department of Immunology, School of Medicine, Shahid Beheshti University of Medical Sciences, Tehran, Iran; Duke University School of Medicine, UNITED STATES

## Abstract

HER2/neu is an immunogenic protein inducing both humoral and cell-mediated immune responses. The antigen-specific cytotoxic T lymphocytes (CTLs) are the main effector immune cells in the anti-tumor immunity. To induce an effective CTL specific response against P5+435 single peptide derived from rat HER2/neu oncogene, we used a liposome delivery vehicle. *In vivo* enhancement of liposome stability and intracytoplasmic delivery of peptides are the main strategies which elevate the liposome-mediated drug delivery. Liposomes containing high transition temperature phospholipids, such as DSPC, are stable with prolonged *in vivo* circulation and more accessibility to the immune system. Incorporation of DOPE phospholipid results in the effective delivery of peptide into the cytoplasm *via* the endocytotic pathway. To this end, the P5+435 peptide was linked to Maleimide-PEG2000-DSPE and coupled on the surface of nanoliposomes containing DSPC: DSPG: Cholesterol with/without DOPE. We observed that mice vaccinated with Lip-DOPE-P5+435 formulation had the highest number of IFN-γ- producing CTLs with the highest cytotoxic activity that consequently led to significantly smallest tumor size and prolonged survival rate in the TUBO mice model. In conclusion, our study indicated that the liposomal form of P5+435 peptide containing DOPE can be regarded as a promising prophylactic anti-cancer vaccine to generate potent antigen-specific immunity.

## Introduction

Since the discovery of tumor-associated antigens (TAAs) in the 1990s, various immunotherapy approaches, such as peptide vaccines, have been developed for cancer treatment [1]. Peptide vaccines are antigenic epitopes derived from TAAs that can sufficiently induce both humoral and cell-mediated immunity promoting the elimination of tumor cells [2]. The peptide vaccines inducing cellular immunity mediated by stimulation of CD8^+^ cytotoxic T lymphocytes (CTLs) are known to be effective for the immunological clearance of solid tumors [3–6].

The human epidermal growth factor receptor 2 (HER2/neu), as a well-known TAA, is overexpressed in ~30% of breast cancers and increases the aggressiveness of tumor cells [6]. Several ongoing clinical trials, such as NCT01570036 and NCT02297698, have shown that antigenic peptides derived from HER2 are effective immunostimulators eliciting a potent immune response that may lead to invaluable clinical outcomes [7], although the ideal HER2-based peptide vaccines are yet under investigation.

Activation of an effective immune response by CTLs occurs *via* uptake of TAAs by dendritic cells, attachment to major histocompatibility complex (MHC) class I molecules, and presentation to CD8 T cells [8]. Hence, sufficient uptake, processing, and the presentation of antigenic peptides by activated antigen-presenting cells (APCs) *via* MHC-I as well as efficient activation of effector cells exert a critical role in promoting an effective anti-tumor immunity [9]. In this regard, to improve the efficiency of peptide vaccines, different approaches including the design of the specific antigenic peptides, application of appropriate antigen delivery systems, and potent adjuvants have been investigated [10–13].

Liposomes, as an efficient nanoparticle delivery system, possess several important advantages as an immuno-adjuvant and delivery platform for vaccines such as biocompatibility, biodegradability, safe, nontoxic and nonimmunogenic products. It has revealed that the physicochemical characteristics of liposomes, such as the lipid content, bilayer fluidity, hydrophobicity, method of antigen attachment, and charge of the particle, dramatically influence the type and potency of the immune response induced by the liposomal vaccines [14–16].

The adjuvant properties of liposomes stem from their interaction with APCs and delivering of antigen and immunostimulators to the APCs [17]. Earlier evidence has shown that liposomes containing the high phase transition temperature (Tc) phospholipids, such as 1,2-distearoyl-sn-glycero-3-phosphocholine (DSPC), are more effective to induce CTL immune response against liposomal peptide than those formulated by low-Tc phospholipids [18, 19].

Dioleylphosphatidylethanolamine (DOPE) is another effective phospholipid for enhancing the efficacy of an antitumor liposomal vaccine. DOPE is a pH-sensitive lipid that mediates the fusion of liposomes and presentation of antigens through the endosomal release of antigens *via* phase transition phenomenon induced in acidic pH of the endosomal compartment [15]. Upon phase transition, DOPE is changed from lamellarity to a hexagonal structure resulting in the fusion of liposome and endosomal membranes. Consequently, the contents of endosomes are released into the cytosol and, thereby, antigens are delivered to MHC class I molecules [20–23].

As demonstrated by our previous studies, two multi-epitope peptides designed from rat HER2/neu, namely P5 and P435, can effectively elicit CTL responses in tumor-bearing mice [4, 6, 24–27]. Although the aforementioned formulations could inhibit tumor growth in mice, the encapsulation efficiency of P5 in cationic liposomes was found to be low. Therefore, in further studies, the P5 peptide was linked to Maleimide-PEG2000-DSPE *via* a covalent bond, which was associated with enhanced incorporation of the peptide into liposomes [6, 28]. It was shown in our previous studies that the conjugated form of P5 and P435 peptides, as a liposomal peptide vaccine could lead to effective CTL responses [29, 30]. In this study, we conjugated two multi-epitope peptides, P5 and P435. The resultant conjugated antigenic peptide (P5+435) was covalently linked to Maleimide-PEG2000-DSPE and then coupled to the surface of prepared liposome formulation composed from DSPC: 1,2-Distearoyl-sn-glycero-3-phosphoglycerol (DSPG): DOPE: Cholesterol. The efficacy of new multi-epitope peptide, P5+435 peptide, in the stimulation of CTL mediated immunity was assessed in the TUBO tumor-bearing mice model.

## Materials and methods

### Animal

BALB/c mice (female, 4–6 weeks) were purchased from the Pasteur Institute (Tehran-Iran) and housed in the vivarium medicine unit at 21°C in 12–12 hour’s light-dark cycles with easy access to ad libitum water and food. This study was approved by the Institutional Ethical Committee and Research Advisory Committee of Mashhad University of Medical Sciences, under the protocol number of 910445 and all animal experiments and methods were carried out in accordance with the relevant guidelines and regulations approved by the ethical committee.

### Cell lines

TUBO, a rHER2/neu protein overexpressing cell line established from a lobular carcinoma that had spontaneously arisen in female transgenic BALB/c mice was kindly provided by Dr. Pier-Luigi Lollini (Department of Clinical and Biological Sciences, University of Turin, Orbassano, Italy). The CT26 murine colon carcinoma cell line (rHER2/neu negative), was purchased from Pasteur Institute (Tehran-Iran). TUBO and CT26 cell lines were cultured in Dulbecco's Modified Eagle's Medium (DMEM) supplemented with 20% fetal bovine serum (FBS) and Roswell Park Memorial Institute (RPMI-1640) medium supplement with 10% FBS, respectively.

### Peptide and biomaterial

P5+435 peptide (ELAAWCRWGFLLALLPPGIAGRRIRGRILHDGAYSLTLQGLGIHGGGC) (P5: ELAAWCRWGFLLALLPPGIAG and P435: IRGRILHDGAYSLTLQGLGIH) containing linker sequence with 95% purity was synthesized and HPLC-purified by Peptron Co (Shanghai, China). DSPC (1,2-Distearoyl-sn-glycero-3-phosphocholine), DSPG (1, 2-Distearoyl-sn-glycero-3-phosphoglycerol), DOPE (Dioleoyl phosphatidyl ethanolamine) and DSPE-PEG2000-Maleimide (Distearoylphsphoethanolamin-N [maleimide (polyethylene glycol)-2000]) phospholipids were purchased from Avanti Polar Lipid (Alabaster, USA). Cholesterol was purchased from Sigma-Aldrich (Steinheim, Germany). All flow cytometry antibodies (CD8, CD4, IFN- γ, and IL-4) and kits and PMA/ionomycin cocktail with brefeldin A were purchased from BD Biosciences (San Diego, USA).

### Linking of P5+435 peptide to Maleimide-PEG 2000-DSPE

Peptide and Maleimide-PEG2000-DSPE with 1.2:1 molar ratio dissolved in DMSO: Chloroform (1:1) were mixed and stirred under argon gas at the room temperature for 48h. To assay the conjugation, thin layer chromatography (TLC) method was done. Hence, peptide, Maleimide-PEG2000-DSPE, and the final product (Peptide + Maleimide-PEG2000-DSPE) were separately spotted on a TLC plate (silica gel 60 F254, Merck, USA). The TLC spotted plate was placed in a chamber containing a mobile phase comprised of Chloroform: Methanol: Water at a volume ratio of 90:18:2. Then, spots were stained with iodine vapor. Thereafter, the DMSO: Chloroform solution was removed using a rotary evaporator (Heidolph, Germany) followed by freeze-drying (VD-800f, Taitech, Japan). The resultant product powder was hydrated with sterile deionized water (pH 7.2) at 30°C to form DSPE-PEG-peptide micelles. The total phospholipids’ content of the final product was determined by the Bartlett phosphate assay method [31]. The linked peptide was quantified indirectly by measuring the fraction of unlinked peptide by using HPLC (High- performance liquid chromatography) method (KNAUER, Berlin, Germany) as presented previously [6]. The conjugation was qualified *via* the Tricine SDS-PAGE method [32]. After quantitative analysis with HPLC the excess peptides were removed *via* three cycle of dialysis against HEPES buffer saline solution (NaCl 150mM, HEPES 10mM, pH 7.2) using a 12–14 kDa MWCO dialysis cassette and then the conjugated peptides were used for post-insertion.

### Preparation and characterization of nanoliposomal formulations

Liposome nanoparticles with a 40 mM total molar ratio were manufactured using the lipid film hydration method. In brief, DSPC: DSPG: Chol: DOPE phospholipids were resolved and mixed in chloroform at a molar ratio of 15:2:3:5, respectively. Chloroform was eliminated by a rotary evaporator at 65°C (Heidolph, Germany). Then, dried lipid film was hydrated with 10 mM, pH 7.2 HEPES buffer containing 5% dextrose and completely dispersed by vortex and bath-sonication at 65°C. The prepared multilamellar vesicles (MLVs) were extruded with a mini extruder (Avestin, Canada) *via* 400, 200, 100 nm polycarbonate membranes to form 100 nm small unilamellar vesicles (SUVs). Liposome formulation (Lip) with the mentioned molar ratio without DOPE was used as a control. The post-insertion of P5+435-PEG-DSPE micelles on the surface of liposome nanoparticles was performed by mixing 100 μg P5+435-PEG-DSPE micelles (based on the linked peptide) and 1ml of liposome nanoparticles incubating at 60°C for 3 h. By this method, the DSPE phospholipid moiety mediates the insertion of the P5+435-PEG-DSPE micelles in the nanoliposome bilayer and the PEG chains expose P5+435 peptides on the outer membrane of nanoliposomes. The nanoparticles size (diameter, nm), surface charge (zeta potential, mV), and polydispersity index (PDI) were analyzed using the dynamic light scattering (DLS) technique (Nano-ZS, Malvern, UK) at the room temperature. All liposomal formulations were stored under argon at 4°C.

### Prophylactic immunization protocol

BALB/c mice were divided into five groups, each group included 9 mice. The liposomal formulation was subcutaneously injected (100μl/mouse) into the left flank three times in bi-weekly intervals (on days 14, 28 and 42 before TUBO tumor challenge (day 0)). The immunized groups were as follows: Free P5+435 peptide, Lip-DOPE, Lip-P5+435, Lip-DOPE-P5+435. The control mice were administrated with HEPES/sucrose buffer. The 10μg dose of free P5+435 peptide and the 5μmol dose of liposome per mouse were applied in each injection. To evaluate immune response, two weeks after the last immunization, three mice of each group were anesthetized with an injection of the ketamine-xylazine solution 20–25 min elapsed before euthanasia for each mouse and scarified by cervical dislocation, and their spleens were collected aseptically. Splenocytes were prepared by passing through 70μm cell strainer and lysis of RBC using ACK lysis buffer. Splenocytes were then used for immunological analysis.

### T cell activation analysis *via* Enzyme-linked Immunospot (ELISpot) assay

To check immune response, T cell activation was assayed *via* evaluation of IL-4 and IFN-γ expression with the ELISpot method according to the manufacturer protocol (U-CyTech, Utrecht, Netherlands). In Brief, 96-well ELISpot plates were coated with each cytokine’s antibodies and incubated overnight at 4°C. Splenocytes of immunized mice were seeded at 3×10^5^ cells in triplicate pre-coated wells and filled with 200μl of P5+435 peptide containing medium for at 37°C 24 h. After the cell’s removal, the biotinylated anti-mouse detection antibodies were added to each well and incubated for at 37°C for 1 h. Finally, spot-forming units (SFU) as the number of IFN-γ and/or IL-4 secreting cells were estimated by counting the number of spots in each well using Kodak 1D image analysis software (Version3.5, Eastman Kodak, Rochester, New York). Results were represented as the number of SFU per 10^6^ cells.

### Intracellular cytokine analysis *via* flow cytometry assay

Intracellular staining of cytokines was done as explained elsewhere [33]. Briefly, RBC-depleted spleen cells were cultured (10^6^ cells/ml) and stimulated with Golgi Plug^TM^ and PMA/Ionomycin cocktail (1μl and 2μl/ml, respectively) at 37°C for 4 h. After stimulation, spleen cells were transferred to flow cytometry tubes (10^5^/tube) and washed with stain buffer (PBS with 2% FCS). Spleen cells were then stained with 1 μl PE-Cy5.5- anti-mouse CD8 or PE-Cy5.5- anti-mouse CD4 antibodies, separately at 4°C for 30 min. After washing with staining buffer, the cells were fixed with Cytofix/Cytoperm^TM^ solution. The fixed cells were washed twice with Perm/Wash^TM^ buffer and stained with (1μl) anti-IFN-γ-FITC and anti-IL-4-PE antibodies at 4°C for 30 min. Splenocytes were also stained with fluorescein isothiocyanate (FITC) and phycoerythrin (PE) Rat IgG2b isotype controls. Following the last wash, 2×10^5^ spleen cells were resuspended in staining buffer (300 μl) and analyzed by BD FACS Calibur™ (BD Biosciences, San Jose, USA).

### *In vitro* CTL assay

Spleen cells, as the effector cells harvested from control and vaccinated mice, were cultured in the presence of 10μg P5+435 peptides and recombinant IL-2 (20U/ml) in 96-well U-bottomed plates for 5 days. TUBO tumor cells and CT26 cells (used as negative control), as the target cells, were resuspended in DMEM-20% FCS were stained with 12.5 μM Calcein AM at 37°C for 1 h in a dark place. Then the effector cells were co-cultured at different ratios with the target cells in triplicate wells in U-bottomed plates. Media containing Triton X-100 was added to the wells seeded TUBO cells to determine the maximum and minimum release. The fluorescence was analyzed by a fluorescent plate reader (FLX 800, Bio-Tek Instrument Inc. USA). The mean percentage of specific lysis was calculated as (release by the CTLs-minimum release by targets)/ (maximum release by targets-minimum release by targets) ×100.

### TUBO tumor challenge

Two weeks after the last immunization, the immunized mice (6 mice per group) were subcutaneously inoculated in the right flank with TUBO tumor cells (5×10^5^ cells/50μl PBS). Three orthogonal diameters (a, b, c) of tumors were measure with a digital caliper. The tumor volume was monitored regularly and calculated according to the formulation [(height × width × length) × 0.5]. The survival and monitoring of tumor volume of mice have followed for 107 days. [(TTE = [log (endpoint)—b] /m, -b = the intercept and m = slope—of the line acquired by linear regression of tumor growth curve) (TGD = TTE of treatment group—TTE of buffer group/TTE of buffer group × 100) (ILS = MST of treatment group/MST of buffer group × 100–100)] were calculated for each groups [34–36]. For ethical consideration, mice were euthanized when the tumor volume was approximately or more than 1000 mm^3^, or their bodyweight drops more than 15% of initial weight. Mice were anesthetized with an injection of the ketamine-xylazine solution, 20–25 min was elapsed before euthanasia for each mouse and sacrificed by cervical dislocation.

### Statistical analysis

All data were statistically analyzed by GraphPad Prism (version 6, San Diego, California). One- and two-way ANOVA and Tukey post hoc tests were used to determine the significant difference among various formulations. Survival data were analyzed by the log-rank (Mantel-cox) test to compare the survival curve. *P*<0.05 were considered to be statistically significant.

## Results

### Conjugation between P5+435 peptide and Maleimide-PEG2000-DSPE

**Fig 1:**
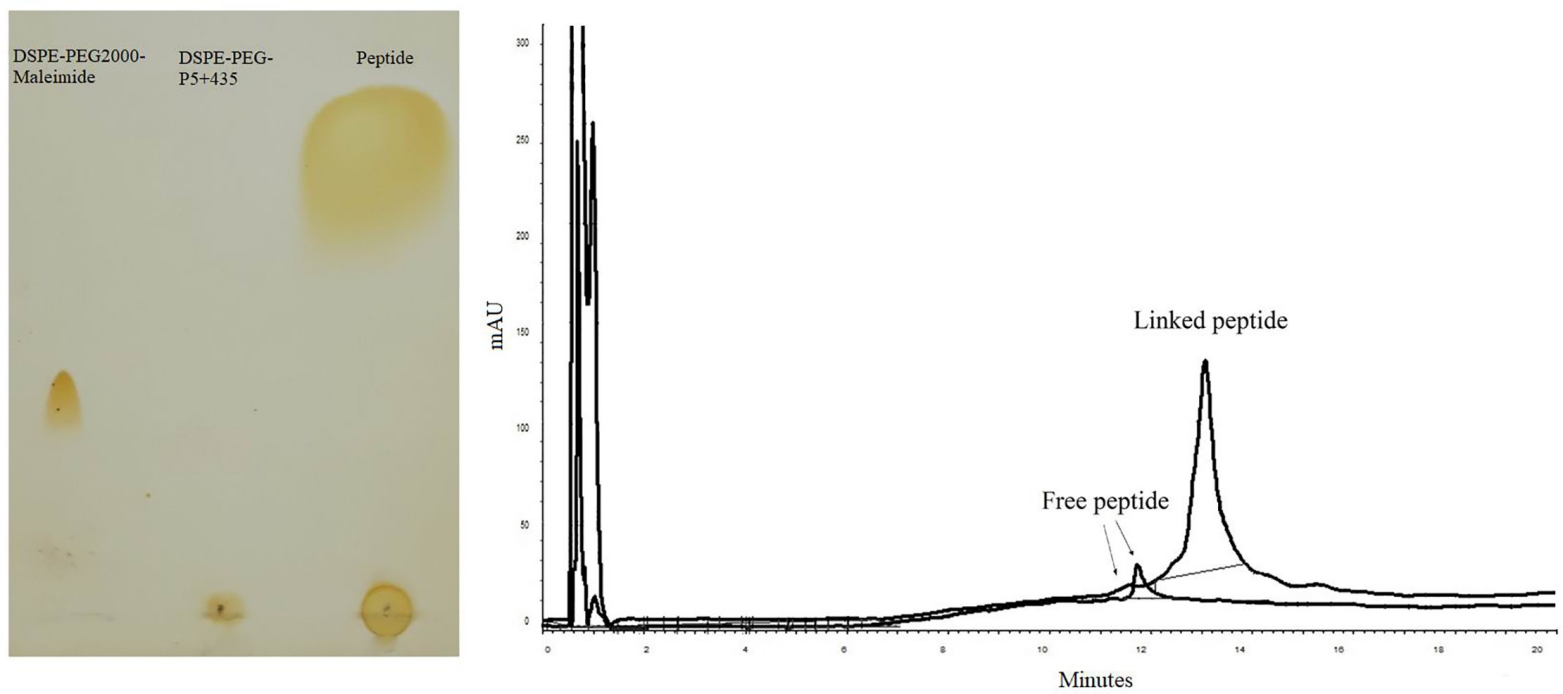
Confirmation of conjugation between P5+435 peptide and Maleimide-PEG-DSPE by using TLC (A) and HPLC (B) analyses.

As confirmed by both chromatography methods, the peptide was successfully linked to Maleimide-PEG2000-DSPE through a suitable linkage between activated maleimide and a thiol group of P5+435 peptide. On the TLC, the disappearance of the Maleimide-PEG2000-DSPE spot shows an almost completed reaction as indicated in Fig 1A. HPLC analysis confirmed that the peptide was linked to the Maleimide-PEG2000-DSPE by ~100% efficiency (Fig 1B).

### Characterization of nanoliposomal vaccine formulations

Table 1 shows the size, PDI, and zeta potential of each liposomal formulation. The size of liposome formulations was ranged from 160 nm to 190 nm. All the formulations showed negative zeta potential. The PDI demonstrated that liposome nanoparticles are completely homogenous (PDI<0.1). Table 1 also shows the amount of phospholipids and the peptide in each formulation.

**Table 1:** Physical properties and the content of phospholipids and P5+435 peptide in each liposomal formulation.

Formulation	Z-average (nm) ± SD	Z potential (mV) ± SD	PDI ± SD	Phospholipid (nmol/μl)	Peptide (ng/μl)
**DSPC-DSPG-Chol-DOPE**	177.3±2.5	-44.4±6.5	0.05±0.02	**-**	**-**
**DSPC-DSPG-Chol-P5+435**	165.8±4.9	-42.5±5.5	0.07±0.036	32.65 ± 2.9	93.13±3.2
**DSPC-DSPG-Chol-DOPE-P5+435**	187.9±2.4	-41.8±5.4	0.1±0.026	41.16 ± 2.3	95.04 ±6.7

### Tricine SDS-PAGE analysis

**Fig 2:**
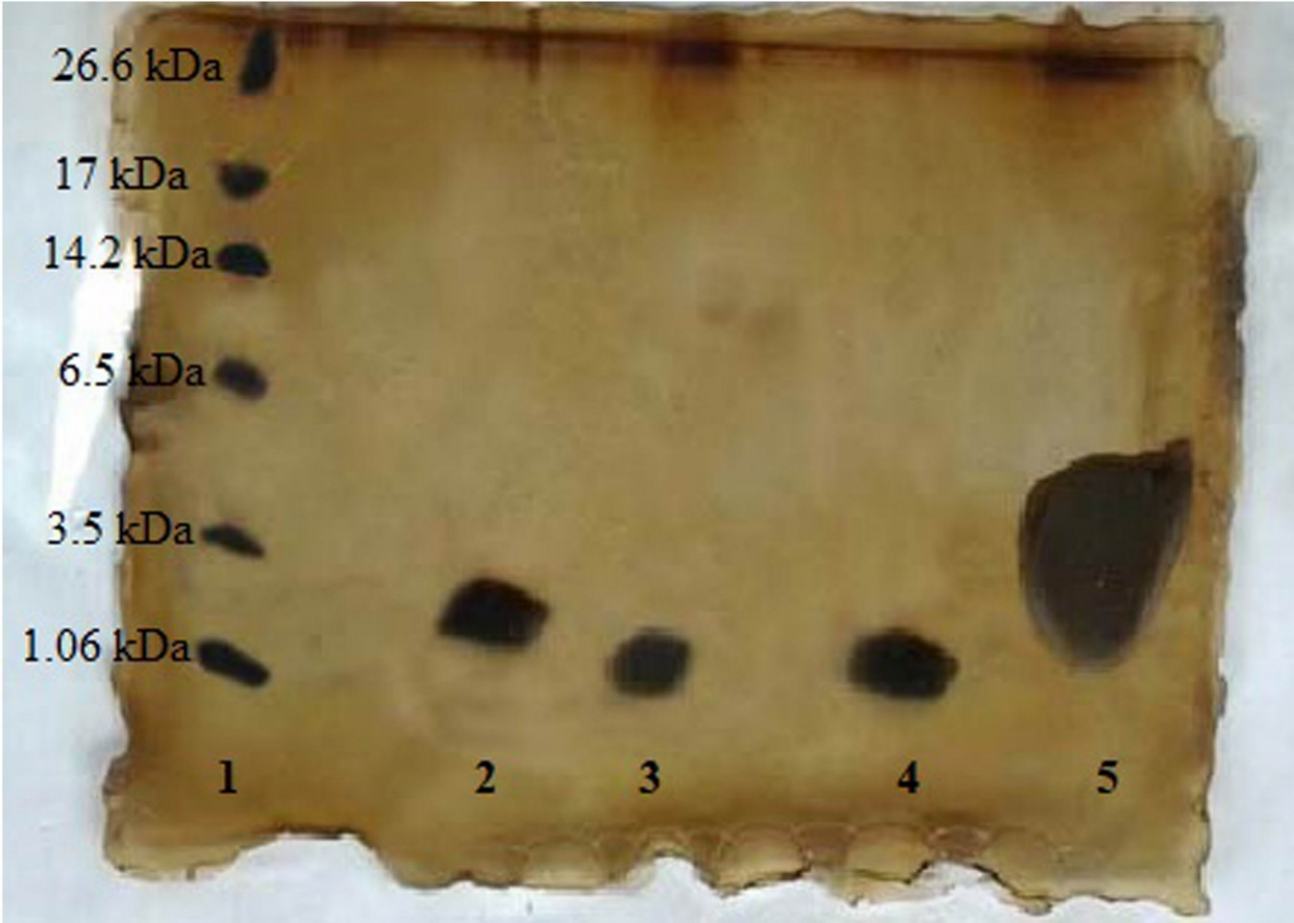
Tricine SDS page analysis. Lane 1: Ladder, Lane 2: Pure P5, Lane 3: Pure P435, Lane 4: P5+435, and Lane 5: Lip-DOPE-P5+435. The peptide concentration of sample was 2 μg/μl.

Qualitative analysis of free P5+435 peptide with Tricine SDS-PAGE revealed a single band (Lane 4) with molecular weight relative to free P5 (Lane 2) and P435 (Lane 3) peptides band. The presence of P5+435-PEG-DSPE in the liposomal formulation was detected (lane 5) with a distinct band similar to the free peptide (Fig 2).

### Extracellular cytokine analysis

**Fig 3:**
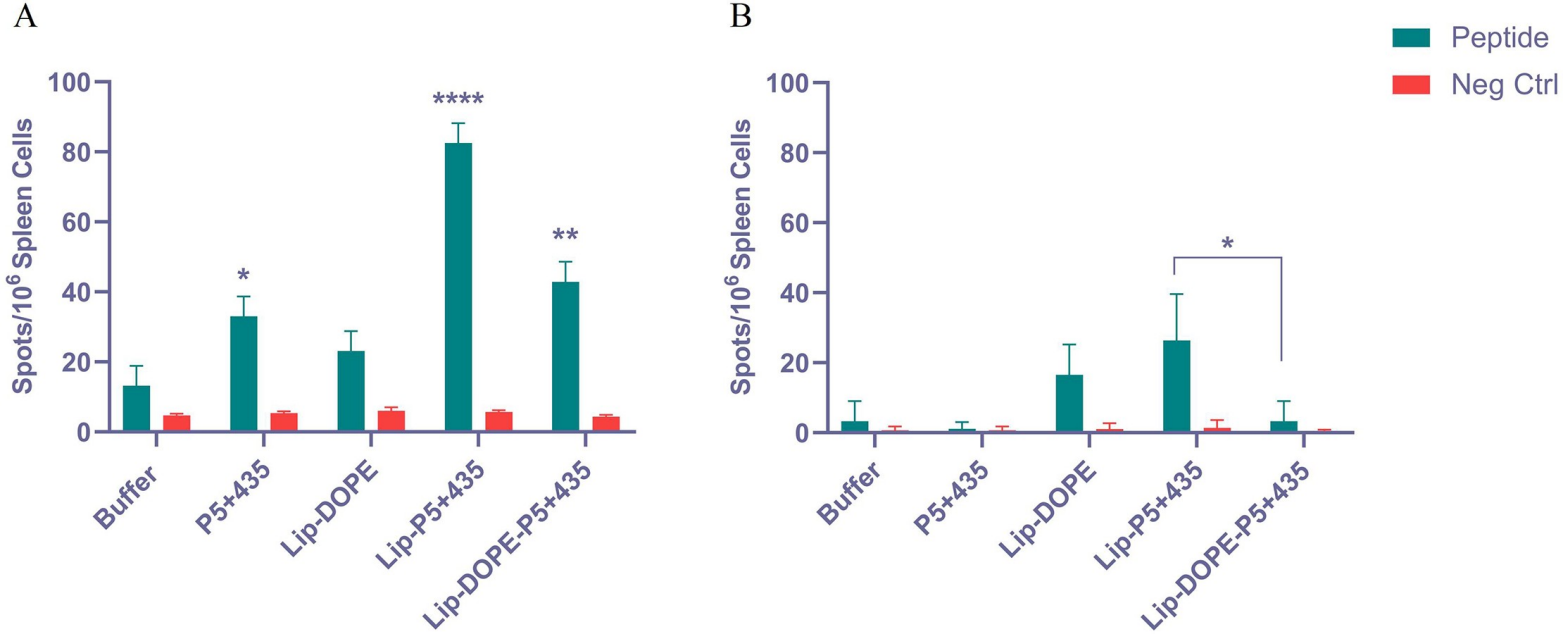
The *In vitro* cytokine production using ELISpot assay. The evaluation of IFN-γ (A) and IL-4 (B) production in P5+435 peptide-stimulated splenocytes isolated from immunized and non-immunized (buffer) BALB/c mice. ****, ** and * denote *P*<0.0001, *P*<0.01 and *P*<0.05, respectively. The data are shown as Mean ± SD (N = 3).

The peptide-specific IFN-γ and IL-4 secretion at the single-cell level of the spleen cells harvested from the mice immunized with different liposomal constructs were evaluated to determine the promotion of cellular immunity response. Of note, Lip-P5+435 was found to significantly induce peptide-specific IFN-γ secretion more than other formulations (P<0.0001). It was also found that Lip-DOPE-P5+435 and P5+435 immunized mice released a significantly higher amount of peptide-specific IFN-γ comparing to the buffer group (P<0.01 and P<0.05, respectively) (Fig 3A). In the case of IL-4 production, Lip-DOPE-P5+435 formulation stimulated considerably lower peptide-specific IL-4 response than Lip-P5+435 (P<0.05) (Fig 3B).

### Intracellular cytokine analysis

**Fig 4:**
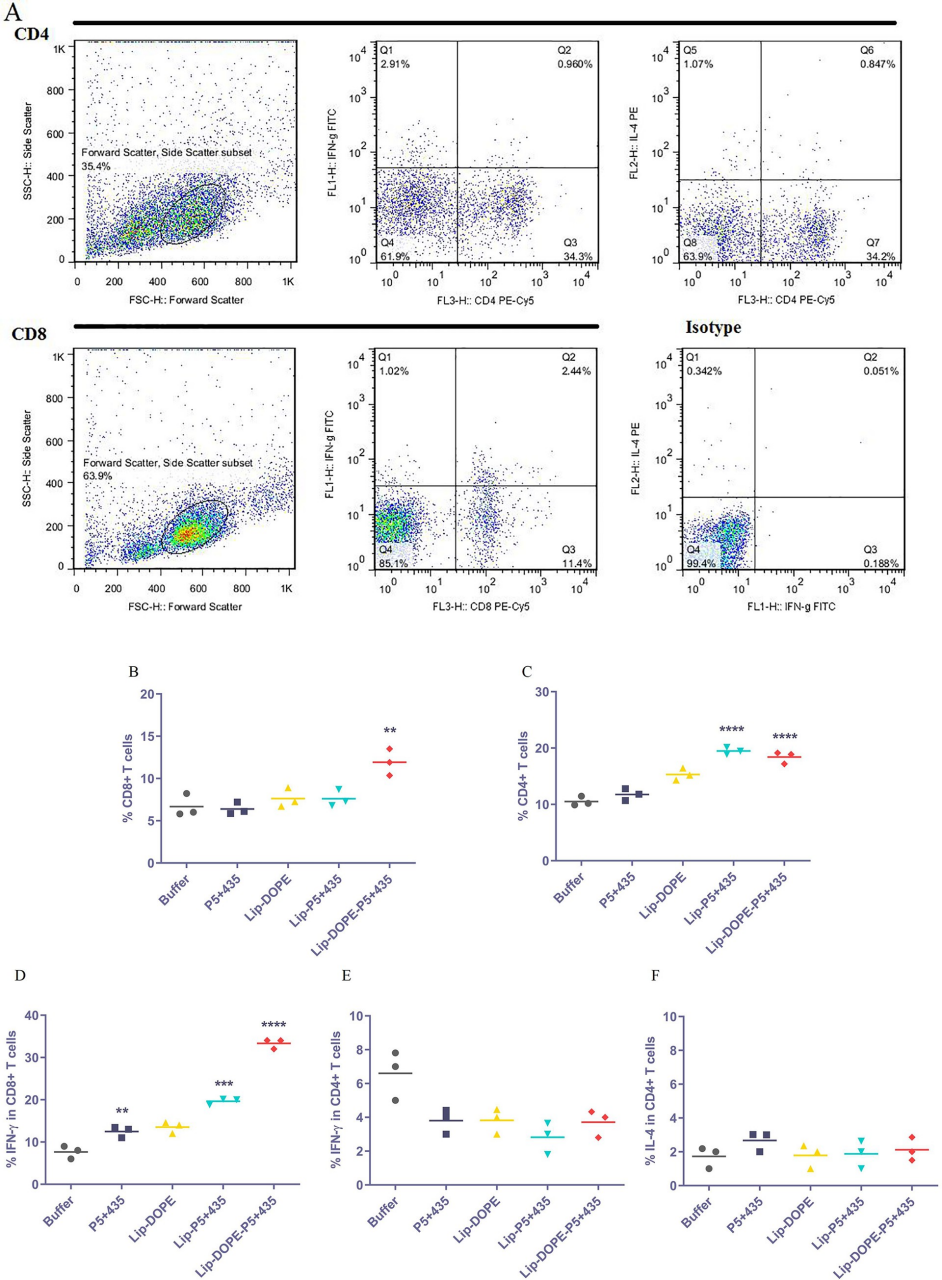
Cytokine analysis by flow cytometric assay. The percentage of cytokine-producing cells in splenocytes of immunized mice was determined by using the depicted strategy of gating (A). The percentage of CD8^+^ and CD4^+^ T cells (B and C, respectively) and IFN-γ in the gated CD8^+^ and CD4^+^ T cells, and IL-4 in the gated CD4^+^ T cells were measured (D, E, and F, respectively). Data represent Mean ± SD (N = 3). **P<0.01, ***P<0.001 and ****P<0.0001.

To evaluated immune response, the levels of cell surface CD markers, CD4 and CD8, and intracellular biomarkers including IFN-γ and IL-4 cytokines were determined by flow cytometry technique in immunized mice spleen cells. The percentage of cytokine-producing cells was measured according to the strategy of gating (Fig 4A). As was shown in Fig 4B, Lip-DOPE-P5+435 had a significantly higher titer of CD8^+^ T cells over Lip-DOPE, Lip-P5+435, and P5+435 groups (P<0.01). In terms of CD4^+^ T cells, both liposomal formulations of the peptide, Lip-P5+435, and Lip-DOPE-P5+435 had enhanced significantly the number of CD4^+^ T cells compared peptide group (P<0.0001) (Fig 4C). Data illustrated that the Lip-DOPE-P5+435 group produced a significantly greater amount of IFN-γ in the CD8^+^ T cell population than all other groups (P<0.0001). In addition, the results also indicated that Lip-P5+435 significantly up-regulated the IFN-γ production by CD8+ T cells in comparison with the P5+435 group (P<0.001) and in P5+435 group compared to the buffer group (P<0.01) (Fig 4D). No significant differences were seen in the results of IFN-γ secretion by CD4^+^ T cells in the different groups (P>0.05) (Fig 4E). The IL-4 production by CD4+ T cells in all groups was low, showing that the shift of T cell-dependent immunity towards Th1 type CD4 T cells in the immunized groups (Fig 4F).

### Cytotoxicity assay

**Fig 5:**
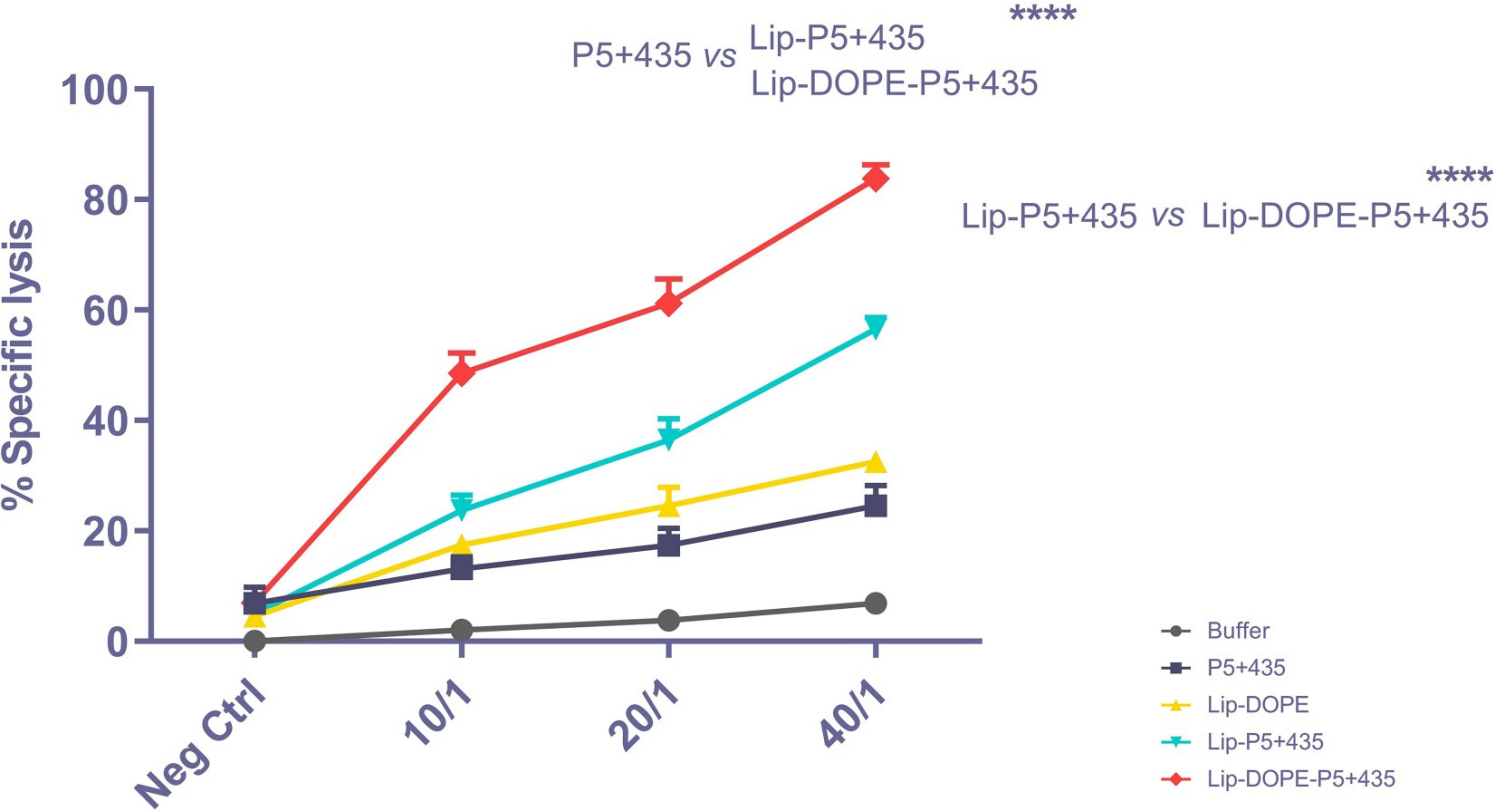
*In vitro* cytotoxic activity of CD8^+^ T lymphocytes. CTLs activity of spleen cells isolated from the immunized mice against rHER2/neu-expressing TUBO cells. Data are shown as Mean ± SD (N = 3). **** Sign shows the significance of difference (*P*<0.0001).

To evaluate the cytotoxic activity of CD8^+^ T cells, TUBO cells were co-cultured with different ratios of spleen cells (40/1, 20/, and 10/1 ratio of effector cells to target cells). The results indicate that a significantly higher cytotoxic activity of CD8^+^ T cells was induced in mice immunized with Lip-DOPE-P5+435 compared to Lip-P5+435 and P5+435 groups and also in Lip-P5+435 compared to P5+435 (P<0.0001) (Fig 5).

### The prophylactic effect of the nanoliposomal formulations in BALB/c mice

**Fig 6:**
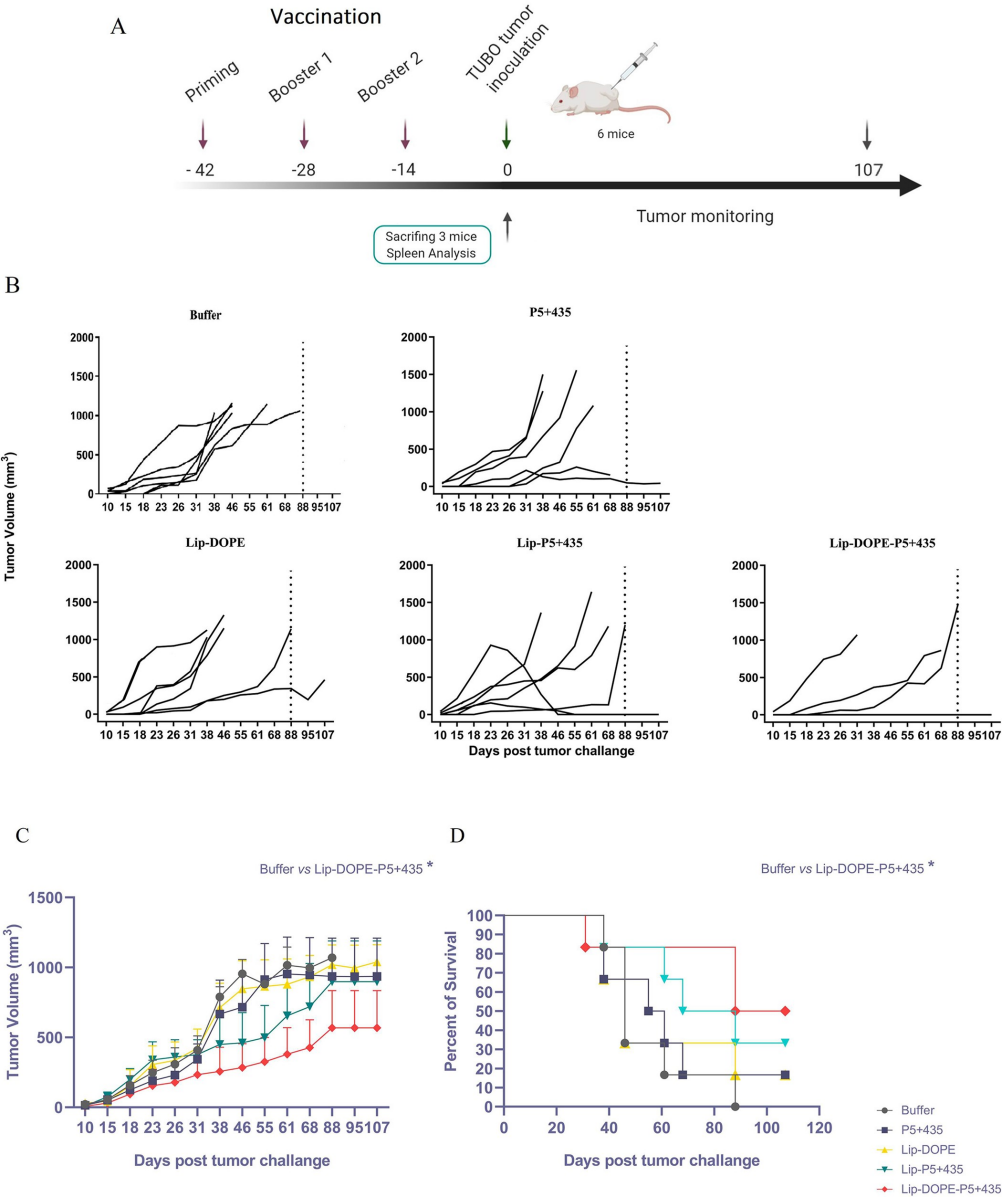
Protective effect of immunization with liposomal formulations in BALB/c mice against rHER2/neu-expressing TUBO cells. (A) Nine mice per group were immunized three times. Two weeks following the last immunization Six mice were subcutaneously challenged with 5×10^5^ TUBO cells. (B) Tumor volume of each mouse per group compared to the buffer group. Dot line shows the last day in which all mice in the buffer group died. (C) Tumor growth was measured two times a week for 107 days. (D) The survival of mice was followed during 107 days. The values are shown as Mean ± SEM (N = 6) *P<0.05.

To evaluate the prophylactic effect of the nanoliposomal formulations, two weeks after the last immunization, 6 mice per group were subcutaneously inoculated with TUBO cells (Fig 6A). Fig 6B shows the individual changes in tumor growth of each mouse in different groups. The result showed that tumor growth of Lip-DOPE-P5+435 immunized mice was significantly slower than P5+435 and two other liposomal groups (P<0.05) (Fig 6C). The median survival time of mice following Lip-DOPE-P5+435 and Lip-P5+435 immunization was higher and the lifespan was increased than P5+435 group. Of note, three mice from Lip-DOPE-P5+435 group and 2 mice from Lip-P5+435 group remained alive till the end of the study (Fig 6D and Table 2). Median survival time (MST), time to reach endpoint (TTE), tumor growth delay (TGD %), and increased life span (ILS%) data for each treatment group are summarized in Table 2.

**Table 2:** Therapeutic efficacy data from different liposomal vaccine formulations in mice bearing TUBO tumor.

Formulation	TTE^a^ (Day ± SD)	TGD^b^ %	MST^c^ (Day)	ILS^d^ %
**Buffer**	49.85 **±** 15.30	-	46	-
**P5+435**	57.76 **±** 30.78	15.86	58	26.08
**Lip-DOPE**	55.14 **±** 33.63	10.62	46	0
**Lip-P5+435**	68.12 **±** 34.28 ^e^	36.65	78 ^e^	69.56
**Lip-DOPE-P5+435**	75.86 **±** 37.22 ^f^^,^ ^g^	52.17	97.5 ^f^^,^ ^g^	111.95

Data are shown as Mean ± SD (N = 6)

a Time to reach end point

b Tumor growth delay

c Median survival time

d Increased life span

e Indicates a significant difference Lip-P5+435 compared with the P5+435 group (*P*< 0.05)

f, g Indicates a significant difference between Lip-DOPE-P5+435 with P5+435 (*P*< 0.01) and Lip-P5+435 (*P*< 0.05) groups.

## Discussion

The present study aimed to evaluate the immunogenicity of liposome-linked P5+435 peptide, an in-silico designed multiepitope long peptide consisting of two CD8^+^ T cells epitopes, P5 and P435, attached to the surface of liposome nanoparticles. It was found that liposomal peptide formulation could significantly induce rHER2/neu-specific CTL mediated immunity. The results are underpinned by another study that shows charged liposomes (positive or negative) provide more robust CTL immune response than neutral vesicles [15]. Of note, it was revealed that liposomes containing DSPC and cholesterol not only induce CD8-mediated immune responses but also provide long-lasting immune response [37]. In mechanism, DSPC phospholipid has been found to increase the stability of liposome formulations, leading to prolonged circulation and more effective delivery of liposomal antigens to dendritic cells and CD8^+^ T cell stimulation [17, 38].

It has been shown that negatively charged liposomes have potent adjuvanticity through activation and also enhancement of antigen uptake by dendritic cells [39]. Moreover, the presence of negatively charged phospholipids, such as DSPG, in the liposome formulation can increase the migration of dendritic cells to the lymph nodes where they meet T cells and induce immune responses [40].

Accordingly, it had been also proved in our earlier studies that the liposomal forms of peptides—either in encapsulated or conjugated form- containing DSPE were superior in eliciting CD8 mediated immunity [6, 25, 29, 30]. Besides, nanoliposomes with high transition temperature phospholipids were also potent in inducing T helper 1 (Th1) cellular responses [18, 25]. Similarly, the Lip-P5+435 could enhance both CD4 and CD8 T cells in the present study.

The pH-sensitive liposomes have been widely used as a drug delivery system for developing prophylactic or therapeutic cancer vaccines. Their effectiveness stems from the capability in delivering small peptides which leads to the induction of efficient immune response and also reduction of their toxicity [41, 42]. Delivering an antigen into the cytosol of dendritic cells is of the most important issues in eliciting cell-mediated immunity, which can be mediated *via* membrane fusion by using fusogenic liposomes [43]. In this way, the use of DOPE-contained nanoliposomes, as a pH-sensitive and fusogenic liposome, is an effective tool for cytosol delivery and assembling of the peptide into the MHC class I pathway, which leads to the activation of CTLs response [15, 20–23]. Our earlier studies show that the inclusion of pH-sensitive lipids, such as DOPE, in the liposomal formulation used as a cancer vaccine can efficiently induce cell-mediated immunity [6, 28–30].

In agreement with our previous studies, an in silico designed HER2/neu peptide, P5+435 possessing MHC I epitopes was sufficiently potent in eliciting cytotoxic activity of CTLs (P<0.0001). The difference between Lip-DOPE and Lip-DOPE-P5+435 also reflected the capability of P5+435 peptide for the induction of antigen-specific CTL responses (P<0.0001). From the liposomal point of view, our results also show that the formulations of the peptide with high transition temperature liposomes -with or without DOPE- significantly enhance the T cell cytotoxic activity over the free peptide, suggesting the role of this form of liposomal formulation in eliciting CTL responses. It was also revealed that the nanoliposomal vaccine with Lip-DOPE-P5+435 formulation could elicit higher specific CTL responses in the TUBO tumor mice model in comparison with Lip-P5+435, indicating a significant role of DOPE fusogenic lipid in the formulation. The resultant effect was found to be accompanied by the most tumor-resistant in mice immunized with Lip-DOPE-P5+435 against TUBO—the HER2/neu expressing- cancer cells and longest survival time. It was found that CD8^+^ T cells isolated from mice immunized with the Lip-DOPE-P5+435 produced more IFN-γ, as an important cytokine in anti-tumor immunity, than other formulations such as Lip-P5+435, further supporting the critical role of DOPE in the formulation. Our results also revealed that the Lip-DOPE-P5+435 formulation increases subpopulations of CD4^+^ T cells as a Th1 profile that contribute to the strong antitumor activity against cancer cells. On the other hand, IL-4, a Th2 cytokine, can suppress Th1-mediated CTL immune response and antigens processing by dendritic cells [44]. Of note, Lip-DOPE-P5+435 formulation induced lower IL-4 production relative to IFN-γ. To sum up, besides the optimal CD8 T cell proliferation and IFN-γ production that were obtained with the Lip-P5+435, the results of the present study show significant *in vivo* efficacy mediated by Lip-DOPE-P5+435 formulation against tumor initiation and progression in TUBO tumor mouse model.

## Concluding remarks

In conclusion, the present study showed the potential of P5+435 peptide for the induction of antigen-specific immunity. Simple liposome formulation with high Tm phospholipid could be an effective vaccine for inducing cell-mediated immunity. Furthermore, the presence of DOPE fusogenic lipid improves the efficacy of a peptide vaccine through the enhancement of intracytoplasmic delivery of peptide that is essential for presentation to MHC class I molecule and induction of antigen-specific CTL immune response. Based on the presented results, in addition to the prophylactic model, this formulation may hold promise for developing a therapeutic vaccine in the HER2 positive breast cancer in the presence of an appropriate adjuvant and/or CD4 restricted peptides such as PADRE.

## Supporting information

S1 File (ZIP)

## Abbreviations

ACK: Ammonium-Chloride-Potassium
APC: Antigen Presenting Cell
Calcein AM: AM, AcetoxyMethyl
CD: Cluster of Differentiation
CTL: Cytotoxic T lymphocyte
DLS: Dynamic Light Scattering
DMSO: Dimethyl sulfoxide
DMEM: Dulbecco's Modified Eagle's Medium
DOPE: Dioleoyl phosphatidyl ethanolamine
DSPC: 1,2-distearoyl-sn-glycero-3-phosphocholine
DSPG: 1, 2-Distearoyl-sn-glycero-3-phosphoglycerol
DSPE: 1,2-Distearoyl-sn-glycero-3-phosphoethanolamine
ELISpot: Enzyme-linked Immunospot Assay
FBS: Fetal Bovine Serum
FDA: Food and Drug Administration
FITC: Fluorescein isothiocyanate
HEPES: 4-(2-hydroxyethyl)-1-piperazineethanesulfonic acid
HER2: Human Epidermal Growth Factor Receptor 2
HPLC: High performance liquid chromatography
ILS: Increased Life Span
Lip: Liposome
MHC: Major Histocompatibility Complex
MLV: Multi Lamellar Vesicle
MST: Median Survival Time
PBS: Phosphate-buffered Saline
PDI: Poly Dispersity Index
PE: Phycoerythrin
PEG: Polyethylene glycol
PMA: phorbol 12-myristate 13-acetate
RBC: Red Blood Cell
RPMI: Roswell Park Memorial Institute
SDS-PAGE: Sodium dodecyl sulfate–polyacrylamide Gel Electrophoresis
SFU: Spot Forming Unit
SUV: Small Unilamellar Vesicle
TAA: Tumor-associated Antigens
TC: Transition Temperature
TGD: Tumor Growth Delay
TLC: Thin Layer Chromatography
TTE: Time to reach End Point
UV: Ultra Violet
