# Supplementary figures and images for: Preparation of nanoliposomes containing HER2/neu (P5+435) peptide and evaluation of their immune responses and anti-tumoral effects as a prophylactic vaccine against breast cancer
Source: PLoS One. 2020 Dec 10;15(12):e0243550. doi: 10.1371/journal.pone.0243550 (PMC7728212; doi:10.1371/journal.pone.0243550)

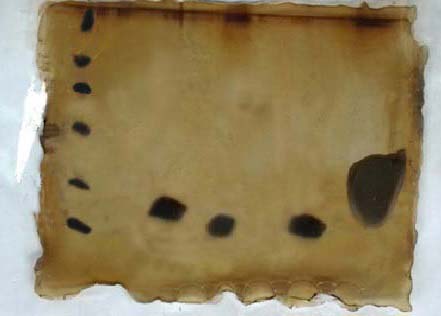

Supplement: S1 File — (ZIP) [file pone.0243550.s001.zip › Supporting Information/Gel.jpg]
